# Effect of synbiotic bread containing lactic acid on blood lipids and apolipoproteins in patients with type 2 diabetes: A randomized controlled trial

**DOI:** 10.1002/fsn3.3039

**Published:** 2022-10-07

**Authors:** Atie Ghafouri, Javad Heshmati, Iraj Heydari, Raheleh Shokouhi Shoormasti, Maria Dulce Estêvão, Ava Sadat Hoseini, Mojgan Morvaridzadeh, Maryam Akbari‐Fakhrabadi, Farnaz Farsi, Mitra Zarrati, Ana Beatriz Pizarro, Farzad Shidfar, Somayeh Ziaei

**Affiliations:** ^1^ Department of Nutrition, School of Public Health Iran University of Medical Sciences Tehran Iran; ^2^ Songhor Healthcare Center Kermanshah University of Medical Sciences Kermanshah Iran; ^3^ Institute of Endocrinology and Metabolism Iran University of Medical Sciences Tehran Iran; ^4^ Immunology, Asthma and Allergy Research Institute Tehran University of Medical Sciences Tehran Iran; ^5^ Universidade do Algarve, Escola Superior de Saúde, Campus de Gambelas Faro Portugal; ^6^ Department of Education and Health Promotion, School of Public Health Iran University of Medical Sciences Tehran Iran; ^7^ Department of Applied Human Nutrition Mount Saint Vincent University Halifax Canada; ^8^ Minimally Invasive Surgery Research Center Iran University of Medical Sciences Tehran Iran; ^9^ Clinical Research Center Fundación Valle del Lili Cali Colombia; ^10^ ICU Department Emam Reza Hospital, Kermanshah University of Medical Sciences Kermanshah Iran

**Keywords:** blood lipids, bread, diabetes, lactic acid, synbiotic

## Abstract

Recently, the use of synbiotics for managing various diseases has dramatically increased. Synbiotics have been shown to be a good approach to influence the composition of the gut microbiota with positive health effects. Management of type 2 diabetes mellitus (T2DM) complications is one of the reasons for the ingestion of synbiotics and so the aim of the current study was to determine the effects of synbiotic bread intake on markers of lipid profile in T2DM patients. One hundred T2DM patients (age between 20 and 60 years) were randomly assigned to four groups to consume different types of synbiotic bread, three times/day, for 8 weeks: “synbiotic + lactic acid” (*n* = 25; IV), “synbiotic” (*n* = 25; III), “lactic acid brad” (*n* = 25; II), or “control” (*n* = 25; I). The measured outcomes included anthropometric characteristics, glycemic control parameters, blood lipids, and apolipoproteins. The consumption of “synbiotic + lactic acid bread” (group IV) and “lactic acid bread” (group II) led to a significant decrease in total cholesterol (TC) and glycated hemoglobin (HbA1c) compared to the “control bread.” The HbA1c levels were also significantly lower when compared to group II. Additionally, apolipoprotein A (Apo A1) levels were significantly decreased in group IV, compared to control and other groups (post hoc analysis). No significant differences between groups were observed for triglyceride (TG), high‐density lipoprotein (HDL), low‐density lipoprotein (LDL), and apolipoprotein B100 (Apo B100) levels. The observed results show that the synbiotic bread (with or without lactic acid) promoted a decrease in total cholesterol (TC) and Apo A1 in diabetic patients when consumed daily for 8 weeks.

## Graphical abstract

synbiotic bread (with or without lactic acid) promoted a decrease in total cholesterol and Apo A1 in diabetic patients when consumed daily for 8 weeks.

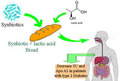

## 1 INTRODUCTION

Type 2 diabetes mellitus (T2DM) is a metabolic disorder characterized by hyperglycemia resulting from insulin resistance or reduced insulin secretion (Lin et al., 2015). The prevalence of T2DM has dramatically increased in the past few decades, and the number of cases worldwide is expected to reach 693 million by 2045 (Cho et al., 2018).

Dyslipidemia, including increased triglycerides (TG), decreased high‐density lipoprotein cholesterol (HDL‐c), and, to some extent, increased low‐density lipoprotein cholesterol (LDL‐c), is a common complication of T2DM (El‐Sayyad et al., 2014). Diabetes combined with dyslipidemia may accelerate the development of atherosclerosis leading to cardiovascular disease (Fan et al., 2018). In diabetes, high insulin levels may cause an increase in LDL‐c which, in turn, may induce the formation of atherosclerotic plaques in arteries and reduce HDL‐c which have anti‐atherogenic properties (El‐Sayyad et al., 2014). It has been reported that more than 75% of diabetic patients die due to cardio‐cerebrovascular diseases annually (Lin et al., 2015). Pharmacologic agents and nutritional therapies are the two main approaches used to prevent and control diabetes mellitus and its complications but dietary modification remains a key player in diabetes management (Barclay et al., 2010).

Recently, the effect of different nutrients on gut microbiota to control the development of metabolic diseases has been widely investigated. It has been reported that changes in gut microbiota composition are related to chronic disorders such as diabetes, dyslipidemia, cardiovascular disease, inflammatory bowel disease, and cancer (Gomes et al., 2014; Shabani et al., 2020). Inappropriate food intake may have a negative impact on intestinal microbiota composition resulting in intestinal permeability and leading to low‐grade inflammation and development of insulin resistance (Bekkering et al., 2013; Kazemi et al., 2020).

The use of probiotics (different strains of microorganisms from different genera including *Lactobacillus*, *Bifidobacterium*, *Bacillus*, among others) or prebiotics (fermentable nondigestible substrates), isolated or in combination (forming synbiotics) (Agah et al., 2020; Markowiak & Śliżewska, 2017), has been widely investigated and considered a good approach to modulate the composition of the gut microbiota favoring the health of the consumers in several different ways, including the management of T2DM complications (Ardeshirlarijani et al., 2019; Vrieze et al., 2012) and contributing to a more efficient management of the metabolic profile, especially lipid profile of T2DM patients (Narmaki et al., 2020; Zarezadeh et al., 2021).

Synbiotics can be composed by probiotics and prebiotics that are recognized for their individual effect on the consumers' health (complementary synbiotics) or have in their composition probiotics specifically chosen to be utilized by the microorganisms present in the mixture (synergistic synbiotics). They may act in different ways, depending on their composition and may enhance the survival of microorganisms present in the host's colon and induce the growth of other probiotic strains (Markowiak & Śliżewska, 2017).

Up to date, synbiotics have mainly been studied in T2DM in the form of supplements and few studies have investigated the effects of synbiotics incorporated in foods such as bread. Bread is one of the most popular foods all over the world and it is considered as a rich‐carbohydrate food item for diabetics. For these reasons, its fortification to control metabolic responses may be a good option. Accordingly, a previous study showed that synbiotic bread intake for 8 weeks decreased triglyceride (TG) and increased HDL‐C in T2DM patients compared to probiotic bread (Shakeri et al., 2014). Another study reported beneficial effects on insulin metabolism in T2DM patients after a daily synbiotic bread consumption for 8 weeks, but the lipid profile was not investigated in this study (Tajadadi‐Ebrahimi et al., 2014).

Moreover, it has also been shown that certain organic acids may regulate metabolic indices in healthy humans, when included in bread‐based meals (Liljeberg et al., 1995). Adding lactic acid to bread ingested by both animals and healthy human subjects has induced beneficial effects on insulin modulation (Fardet et al., 2006; Östman et al., 2005). However, the direct effect of lactic acid added to bread on the lipid profile of T2DM patients has not been studied so far and there is limited evidence regarding the effect of synbiotic foods on the lipid profile of T2DM patients. Thus, this is the first study which explored the effects of different types of bread, either incorporated with a synbiotic mixture, lactic acid, or a combination of both, on the lipid profile of T2DM patients.

## 2 METHODS

### 2.1 Study design and subjects

The current randomized, double‐blinded, controlled clinical trial was carried out between March and December in 2016. The participants were selected from the Firouzgar Clinic of Endocrinology and Metabolism of the Iran University of Medical Sciences, Tehran, Iran. The definite diagnosis of diabetes (Resnick et al., 2000) was performed by a single expert endocrinologist based on the American Diabetes Association criteria: fasting glucose ≥126 mg/dl; postprandial (2‐h) glucose ≥200 mg/dl; and hemoglobin A1c ≥ 6.5%. The inclusion criteria for the participants were body mass index (BMI) between 21 and 59 kg/m^2^, age between 20 and 60 years, using glucose‐lowering drugs, and a history of DM > 6 months. The predefined exclusion criteria were being pregnant or breastfeeding, using insulin therapy, consumption of alcohol, smoking, taking pre‐ or probiotics, antibiotics, multivitamins, or any dietary supplements at the time of recruitment, history of liver or kidney diseases, malignant tumors, metabolic, cardiovascular, or infectious diseases, hypertension, thyroid dysfunctions, gastrointestinal diseases, and being a professional athlete. All the eligible subjects took part in the trial voluntarily and signed an informed consent before entering the study. The trial protocol was approved by the Ethics Committee of Iran University of Medical Sciences, Tehran, Iran (ethics code; IR.IUMS.REC.1394.26524). In addition, this research was registered in the Iranian Registry of Clinical Trials (IRCT no. IRCT201505242709N33), which can be accessed on the IRCT website.

### 2.2 Sample size

The required sample size of the study was calculated using the mean difference of serum insulin obtained from a similar randomized controlled trial (RCT) (Ghafouri et al., 2019), considering the type 1 error as 5% (α = 0.05), type 2 error as 20% (β = 0.2; power = 80%), and 20% dropout. Based on this calculation, a total of 100 T2DM patients (25 individuals in each group) were needed for this project.

### 2.3 Randomization and intervention

The 100 patients were randomly assigned with an allocation ratio of 1:1 to one of the four treatment groups (A, B, C, and D) by an independent assistant not involved in the study using the balanced block randomization procedure, in blocks stratified by sex and body mass index (BMI < 30 kg/m^2^ and BMI ≥ 30 kg/m^2^). All researchers, participants, and the statistician were blinded on the intervention allocation. We have selected lactic acid as a separate group for increasing digestibility and intake rate of nutrients.

Patients in Group I received the “control bread” containing 3 g of beta‐glucan; Group II received the “lactic acid bread” containing 3 g of beta‐glucan and 4 g of lactic acid; Group III was assigned to ingest the “synbiotic bread” which included beta‐glucan (3 g), *Bacillus coagulans* (1 × 10^8^ CFU), and inulin (10 g); and Group IV received the “synbiotic+lactic acid bread” containing beta‐glucan (3 g), *B. coagulans* (1 × 10^8^ CFU), inulin (10 g), and lactic acid (4 g). The amount mentioned for each prebiotic and for the probiotic corresponds to a package of 120 g bread, which was the dose ingested per day. All participants were asked to consume the assigned bread, 3 times a day (3 × 40 g bead per day), during an 8‐week intervention period. Beta‐glucan was incorporated in all types of bread to keep a similar color and to maintain the taste of high fiber bread in all bread types.

The Forni Bread Company, Tehran, Iran provided all types of bread. The extraction of inulin from oat bran and the separation of beta‐glucan from barley were carried out by the Iran Tejarat Company. The preparation of *B. coagulans* was carried out by the Zist Takhmir Pharmaceutical Company (Tehran, Iran). Administration of *B. coagulans* with carbohydrates and protein has been demonstrated to elevate absorption of these macronutrients and can also promote intestinal digestion (Cao et al., 2020; Jäger et al., 2018). The taste, color, and smell of breads were the same in all groups. The bread has been made in as low a temperature as possible in industrial method that has been approved previously (Penhasi et al., 2021; Thang et al., 2019).

All four types of bread had the following composition (per 100 g): 47.2 g carbohydrates, 9.2 g protein, 4.0 g fat, 37.2 moisture, and 2.4 ash, providing 260 Kcal of energy. Breads were all packaged with the same specific packages labeled with four codes (A, B, C, and D) by a third person to make sure that the researchers and participants were blinded in relation to the type of intervention until the end of the study. To verify and facilitate adhesion to the intervention, a 1‐week supply of the assigned bread was given to each participant each week. Additionally, one of the researchers contacted the participants every week to carry out a telephone interview to try to reduce dropouts.

Participants were asked to maintain their usual dietary intakes, physical activity, and lifestyle habits during the 8‐week intervention. They were also asked not to consume any other type of bread besides the one given by the researchers. Participant's dietary intake was assessed by collecting 24‐h food recalls for three nonsequential days at the onset and the end of the protocol and the Nutritionist IV software (First Data Bank; Hearst Corporation, San Bruno, CA, USA) was used for analyzing recall's data. Physical activity of participants has been evaluated through the International Physical Activity Questionnaire (IPAQ).

To examine anthropometric characteristics, the measurements of body weight, height, and BMI (weight in kg divided by height in m^2^) were recorded at the beginning and after the intervention period. For this purpose, weight and height were measured twice to an accuracy of 0.1 kg and 0.5 cm, with no shoes and with light clothes, using a Seca scale (Seca, Hamburg, Germany) and a stadiometer (Seca), respectively. For measuring the biochemical variables, a sample (10 cc) of venous blood samples was collected from all subjects on an overnight fasting at 08.00 h at Firouzgar Laboratory of Endocrine and Metabolism Centre, also at the beginning and after the intervention period. Blood samples were centrifuged at 1008*g*‐force for 10 min, at 4 °C to separate serum. Then, serum samples were held in a freezer at −80°C until the biochemical analysis was carried out.

In this RCT, we evaluated the lipid profile as primary and apolipoprotein A1 (Apo A1) and apolipoprotein B100 (Apo B100) as secondary outcomes. The levels of total cholesterol (TC), triacyglycerol (TG), high‐density lipoprotein cholesterol (HDL‐c), and low‐density lipoprotein cholesterol (LDL‐c) in serum were detected through enzymatic colorimetric assays (Pars Azmoun, Tehran, Iran) on an automated clinical chemistry (ACE) analyzer (Sciapparelli Biosystems Inc., PA, USA) by a single laboratory operator who was uninformed about the assigned intervention for all outcomes. Apo A1 and Apo B100 were evaluated through the Immunoturbidimetric assay (Pars Azmoun, Tehran, Iran).

### 2.4 Statistical analysis

All statistical analyses were performed using SPSS software version 22 (IBM, New York, NY, USA). The normality of quantitative data distribution was evaluated with the Kolmogorov–Smirnov test. Data with normal and non‐normal distribution are presented as means ± standard error of the mean (SEM) and median (25th and 75th percentiles), respectively.

One‐way analysis of variance (ANOVA) or Kruskal–Wallis test was used for determining the difference of variables between groups. If there is a significant within‐group difference, the post hoc method was carried out using Tukey's test. Furthermore, analysis of covariance (ANCOVA) was performed to adjust in terms of baseline values, age, weight, and BMI of parameters in the two groups. A *p*‐value <.05 indicates a statistically significant result.

## 3 RESULTS

**FIGURE 1:**
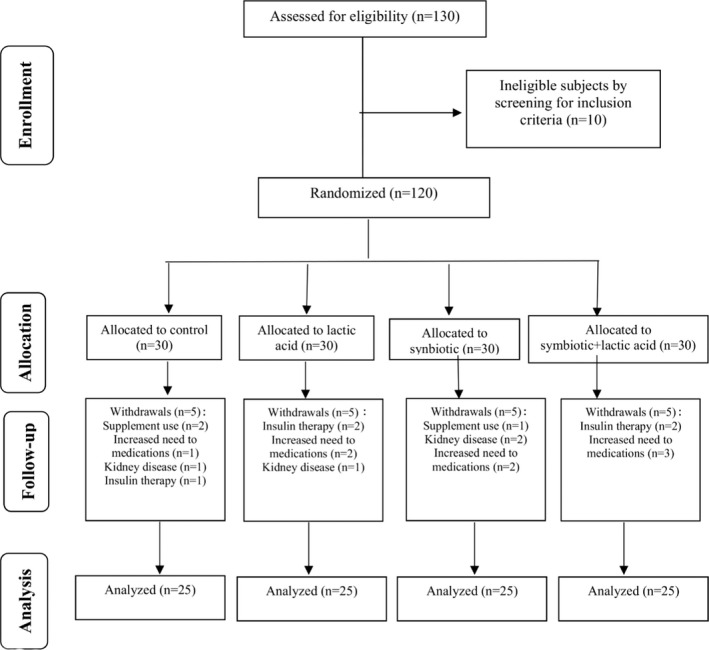
Flow diagram of the study process 2019

From the 130 subjects screened for participation in the trial, only 120 were eligible, interested in participating, and were initially enrolled. Later, 20 subjects were excluded due to the following reasons: withdrawals, using supplements, the increased need for medications, kidney disease, and insulin therapy. Of the 100 individuals remaining (57 males and 43 females), 75% were randomized to the three intervention groups, lactic acid bread (Group II, *n* = 25), synbiotic bread (Group III, *n* = 25), or synbiotic + lactic acid bread (Group IV, *n* = 25) and 25% to the control group (Group I, *n* = 25). The flow diagram of the study is presented in Figure 1. No serious side effects were reported by the participants following the consumption of the supplied bread.

Table 1 presents the age and anthropometric parameters of the study participants. There were no significant differences between the study groups in terms of age, gender, and body mass index (BMI). There were also no significant differences between groups in relation to the dietary intake, at the baseline of the trial (Table 2). There was also no significant difference between study groups in regard to physical activity.

**TABLE 1:** General characteristic of study participants 2019

Variable	Study groups				
	I: Control bread (*n* = 25)	II: Lactic acid bread (*n* = 25)	III: Synbiotic bread (*n* = 25)	IV: Synbiotic + lactic acid bread (*n* = 25)	*p*‐value^a^
Age (y)	54.60 ± 0.83	55.00 ± 0.97	54.92 ± 1.02	53.88 ± 1.09	.84
Gender men (%)/women (%)	15 (63)/10 (40)	13 (52)/12 (48)	16 (64)/9 (36)	13 (52)/12 (48)	.77
BMI at study baseline (kg/m^2^)	27.04 ± 0.50	26.33 ± 0.46	26.39 ± 0.51	26.83 ± 0.42	.67
BMI change (kg/m^2^)	−0.44 ± 0.19	−0.05 ± 0.14	−0.68 ± 0.24	−0.52 ± 0.18	.14

*Note*: All values are mean ± SEM.

Abbreviation: BMI, Body mass index.

^a^ Obtained from one‐way analysis of variance (ANOVA).

**TABLE 2:** Dietary intake of study participants after 8 weeks of intervention

Variable	Study groups	Baseline	End‐of‐trial	*p*‐value^a^
Energy (kcal/day)	I: Control bread	2203.21 ± 81.02	2192.00 ± 70.79	.87
	II: Lactic acid bread	2094.62 ± 58.68	2130.44 ± 59.14	
	III: Synbiotic bread	2212.38 ± 81.17	2138.17 ± 56.97	
	IV: Synbiotic+ lactic acid bread	2085.71 ± 57.79	2130.28 ± 58.66	
Total fat (g/day)	I: Control bread	81.84 ± 3.36	80.48 ± 2.84	.84
	II: Lactic acid bread	79.72 ± 2.09	78.28 ± 2.41	
	III: Synbiotic bread	82.16 ± 3.19	79.44 ± 2.62	
	IV: Synbiotic+ lactic acid bread	79.70 ± 2.09	77.23 ± 13.79	
PUFA (g/day)	I: Control bread	11.27 ± 2.43	11.28 ± 1.39	.75
	II: Lactic acid bread	11.72 ± 1.56	11.25 ± 2.25	
	III: Synbiotic bread	11.43 ± 2.56	11.72 ± 1.35	
	IV: Synbiotic+ lactic acid bread	11.52 ± 1.34	11.24 ± 2.11	
Vitamin E (g/day)	I: Control bread	4.49 ± 0.88	4.94 ± 0.82	.07
	II: Lactic acid bread	4.94 ± 0.80	4.94 ± 0.86	
	III: Synbiotic bread	4.49 ± 0.88	4.72 ± 0.79	
	IV: Synbiotic+ lactic acid bread	4.82 ± 0.80	4.38 ± 0.87	
Se (g/day)	I: Control bread	0.049 ± 0.010	0.049 ± 0.001	.06
	II: Lactic acid bread	0.044 ± 0.001	0.046 ± 0.001	
	III: Synbiotic bread	0.043 ± 0.001	0.049 ± 0.001	
	IV: Synbiotic+ lactic acid bread	0.034 ± 0.001	0.046 ± 0.001	
Fiber (mg/day)	I: Control bread	14.27 ± 0.53	14.58 ± 0.47	.75
	II: Lactic acid bread	15.14 ± 0.67	14.60 ± 0.75	
	III: Synbiotic bread	13.32 ± 0.60	14.19 ± 0.49	
	IV: Synbiotic+ lactic acid bread	15.10 ± 0.63	15.06 ± 0.50	

*Note*: All values are mean ± SEM.

Abbreviations: PUFA, Polyunsaturated fatty acid; TDF, Total dietary fiber.

^a^ Obtained from one‐way analysis of variance (ANOVA).

**FIGURE 2:**
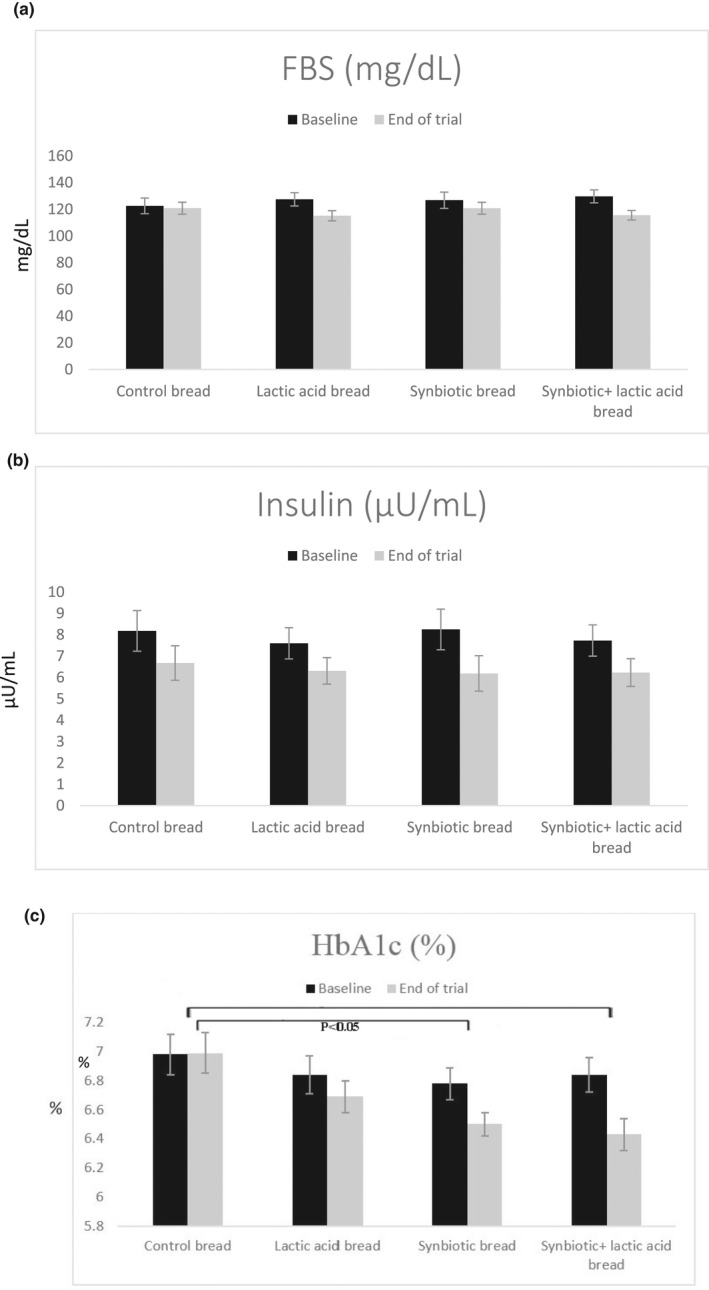
Means ± SEM of Fasting Blood Sugar (FBS) (a), Insulin (b), and Glycated Hemoglobin (HbA1c) (c) in four study groups recorded at the baseline and the end of the trial

The effect of the intervention with the different types of synbiotic bread on glycemic control parameters is presented in Figure 2. The results show that the glycated hemoglobin (HbA1c) percentage has decreased significantly in the “synbiotic + lactic acid bread” group (IV) and in the “synbiotic bread” (group III) compared to the control and the “lactic acid bread” (groups I and II, respectively). No significant differences were observed between the four groups for Fasting Blood Sugar (FBS) and insulin in baseline and end of the trial.

Table 3 compares the changes in lipid profile and apolipoprotein levels between the groups after 8 weeks of intervention. At the end of the trial, total cholesterol (TC) and triglyceride (TG) decreased significantly compared to the baseline (*p* = .03 and *p* = .001, respectively) in the “synbiotic + lactic acid bread” group (IV), and LDL‐c increased significantly compared to the baseline (*p* = .002), while there were no significant changes in HDL‐c. Also, after the intervention, TG decreased significantly (*p* = .007) in the “synbiotic bread group” (III) compared to the baseline values.

**TABLE 3:** Comparison of lipid profile outcomes between the study groups at baseline and at the end of the intervention

Variable	Study groups	Baseline	End‐of‐trial	P_2_ ^a^	Mean difference^b^
TC (mg/dl)	I: Control bread	157.28 ± 29.4	155.68 ± 27.3	0.11	18.40 ± 34.07
	II: Lactic acid bread	153.72 ± 30.8	136.24 ± 24.4	0.39	−17.55 ± 24.11
	III: Synbiotic bread	175.36 ± 27.9	156.92 ± 23.2	0.01	−19.44 ± 37.81
	IV: Synbiotic+ lactic acid bread	174.84 ± 33.7	154.04 ± 3.03	0.03	−20.80 ± 44.43
	P_1_ ^c^	0.10	0.03		0.001*
HDL‐c (mg/dl)	I: Control bread	46.60 ± 8.51	43.96 ± 8.82	0.21	3.36 ± 13.02
	II: Lactic acid bread	45.32 ± 9.76	45.21 ± 9.76	0.13	3.86 ± 12.21
	III: Synbiotic bread	44.72 ± 8.93	42.48 ± 8.55	0.24	−2.24 ± 9.29
	IV: Synbiotic+ lactic acid bread	43.04 ± 8.68	42.80 ± 9.21	0.91	−0.24 ± 10.58
	P_1_ ^c^	0.35	0.93		0.18
LDL‐c (mg/dl)	I: Control bread	83.59 ± 19.63	85.03 ± 27.29	0.82	1.44 ± 31.29
	II: Lactic acid bread	78.59 ± 22.68	78.58 ± 17.66	0.99	5.35 ± 25.47
	III: Synbiotic bread	88.95 ± 26.47	79.51 ± 16.90	0.08	−9.44 ± 26.22
	IV: Synbiotic+ lactic acid bread	68.29 ± 25.28	80.67 ± 18.52	0.002	−17.62 ± 25.42
	P_1_ ^c^	0.69	0.06		0.06
TG (mg/dl)	I: Control bread	161.0 ± 70.07	43.96 ± 8.82	0.91	−11.12 ± 49.96
	II: Lactic acid bread	155.36 ± 85.17	122.64 ± 58.28	0.09	−17.61 ± 51.75
	III: Synbiotic bread	171.36 ± 34.13	144.64 ± 40.74	0.007	−26.72 ± 45.43
	IV: Synbiotic+ lactic acid bread	166.43 ± 67.26	142.20 ± 49.72	0.001	−24.24 ± 32.37
	P_1_ ^c^	0.20	0.22		0.22
Apo A1 (mg/dl)	I: Control bread	148.48 ± 21.41	141.12 ± 43.60	0.19	−07.04 ± 26.27
	II: Lactic acid bread	130.12 ± 44.66	111.84 ± 23.54	0.03	−19.28 ± 28.61
	III: Synbiotic bread	103.04 ± 26.05	91.52 ± 22.44	0.001	−13.00 ± 26.73
	IV: Synbiotic+ lactic acid bread	88.80 ± 16.88	77.6 ± 16.36	0.001	−53.48 ± 23.93
	P_1_ ^c^	0.31	0.24		0.001**
Apo B100 (mg/dl)	I: Control bread	86.44 ± 27.66	88.52 ± 23.10	0.71	−0.92 ± 12.30
	II: Lactic acid bread	106.52 ± 27.98	96.00 ± 21.72	0.03	−10.43 ± 24.89
	III: Synbiotic bread	106.34 ± 31.32	112.21 ± 28.31	0.03	−16.52 ± 36.33
	IV: Synbiotic+ lactic acid bread	108.22 ± 24.44	99.34 ± 21.22	0.005	−11.20 ± 17.90
	P_1_ ^c^	0.10	0.07		0.14

*Note*: Values are presented as mean ± SEM.

Abbreviations: TC, Total cholesterol; TG, Triglyceride; HDL‐c, High‐density lipoprotein cholesterol; LDL‐c, Low‐density lipoprotein cholesterol; Apo A1, Apolipoprotein A1; Apo B100, Apolipoprotein B100.

^a^ *p*‐values represent the differences between the study groups after 8 weeks of intervention obtained from one‐way analysis of variance (ANOVA) or Kruskal–Wallis test.

^b^ Mean difference within groups.

^c^ *p*‐value represents the changes in metabolic parameters between the study groups obtained from analysis of covariance (ANCOVA) adjusted based on age, weight, body mass index (BMI), and baseline values of variables.

*Significant differences between Groups I and III (*p* = .008) and Groups I and IV (*p* = .009); **Groups II and IV (*p* < .001); Groups I and IV (*p* < .001); and Groups II and III (*p* < .001).

Consumption of the synbiotic + lactic acid bread induced a significant decrease in total cholesterol (TC) compared to the consumption of the control bread (−20.80 ± 44.43 and 18.40 ± 34.07, respectively; *p* < .001) and a similar result was observed between the lactic acid bread and the control (−19.44 ± 37.81 and −18.40 ± 34.07, respectively; *p* = .001). In the adjusted model, considering age, weight, BMI, and baseline values, these findings for TC remained significant.

Our results also indicated that Apo A1 and Apo B100 levels significantly decreased after the intervention in all three intervention groups (II, III, and IV) compared to their respective baselines. However, comparing the changes between the groups, the results indicate that Apo A1 levels significantly decreased in the “synbiotic + lactic acid bread” (IV) compared to the control and to the other groups (post hoc analysis results *p* < .05), but there were no significant differences between the groups for Apo B100 levels.

## 4 DISCUSSION

The present randomized clinical trial was carried out to evaluate the effect of bread prepared with synbiotic (which may be classified as a complementary synbiotic (Swanson et al., 2020) as it was prepared including *B. coagulans* (a probiotic) and inulin (a prebiotic), and we may consider that both components may have independent beneficial effects on consumers) and/or lactic acid on lipid profile and apolipoprotein levels on patients with T2DM. Our results show that the levels of TG, LDL‐c, HDL‐c, and Apo B100 did not change significantly between groups, while TC and Apo A1 levels were significantly decreased in “synbiotic + acid lactic bread” group (group IV) compared to the control group (I). Our result has endorsed previous evidence about beneficial effects of probiotic supplementation on metabolic parameters in nonalcoholic fatty liver disease (NAFLD) patients (Saleh‐Ghadimi et al., 2021).

Probiotics (different strains of microorganisms) and prebiotics (several types of indigestible fermented substrates) are supplements that may be added to food or administered by other ways (for example, in capsules), isolated or combined as synbiotics, with the main objective of regulating gut microbiota with positive effects on the host health (Konuray & Erginkaya, 2018a; Ooi & Liong, 2010).

A recent meta‐analysis showed that synbiotic supplementation may result in an improvement in triglycerides (TG) and total cholesterol (TC) plasma levels in diabetic patients (Tabrizi et al., 2018). It has been proposed that the ingestion of synbiotic ingredients may modulate the lipid profile values by several mechanisms including: (1) the production of short‐chain fatty acid (SCFA) and other molecules, such as carbon disulfide and methyl acetate; (2) the regulation of lipid synthesis, for example, by inhibiting the enzymes involved in cholesterol synthesis; (3) by facilitating cholesterol elimination through feces; (4) through the assimilation of cholesterol, as cholesterol may bind to the bacterial cellular surface (for example, in *Lactobacillus acidophilus*); (5) by interfering with bile salts' recycling; (6) the disruption of cholesterol micelles; (7) the deconjugation of bile salt; and (8) by the bacterial bile salt hydrolase activity (Anandharaj et al., 2015; Lee et al., 2009; Lye et al., 2010).

Several studies, including randomized controlled trials (RCTs), carried out both in different animal models (with high similarities with humans regarding cholesterol and bile acid metabolism) and humans (both healthy and with different types of dyslipidemia or other diseases) resulted in controversial findings, with some suggesting that prebiotic, probiotics, and synbiotics may induce significant decreases in total cholesterol, LDL‐c, and/or triglycerides and also increases in HDL‐c (e.g., (Hadi et al., 2019; Kießling et al., 2002; Rajkumar et al., 2015)) and others, concluding that these supplements do not affect lipid profiles or just induce increments in HDL‐c levels (e.g., (Greany et al., 2008; Nabhani et al., 2018; Razmpoosh et al., 2019; Tajabadi‐Ebrahimi et al., 2017)).

In general, our results (Table 3) revealed that the ingestion of the “lactic acid bread” (by group II) induced a slight but significant decrease in both Apo A1 and Apo B100 levels when compared to baseline. The Apo A1 apoprotein is the major protein component in HDL while Apo B100 is present in low‐density lipoprotein (LDL) and very low‐density lipoprotein (VLDL) and both apoproteins may be considered as markers for the presence of these lipoproteins in the plasma (Morita, 2016; Su & Peng, 2020). Additionally, and comparing with the values registered at the baseline, the ingestion of the “synbiotic bread,” containing *B. coagulans* and inulin (group III), induced significant decreases in total cholesterol (TC), triglycerides (TG), and Apo A1 levels and also a significant increase in Apo B100, while the ingestion of the “synbiotic+lactic acid bread” (group IV) induced significant decreases in TC, TG, Apo A1, and Apo B100 and a significant increase in LDL‐c concentrations in the plasma within groups.

The most studied bacteria *(Lactobacillus*, *Bifidobacterium*, and some species of *Saccharomyces*) used as probiotic are lactic acid‐producing species that do not tolerate high temperatures, while *B. coagulans* is a lactic acid‐producing and a spore‐forming species, and its spores may resist high temperatures (at least up to 115°C) (Janštová & Lukášová, 2001). Despite the fact that this temperature is lower than the usual bread baking temperatures, some authors consider *B. coagulans* as a better option to use in processed foods such as bread (Konuray & Erginkaya, 2018a; Lee et al., 2019), as is the case in the present trial. Although there may be some risk associated with the use of *Bacillus* species as probiotics due, for example, to their capacity of producing coagulin that may negatively affect enteric microorganisms, *B. coagulans* has been Generally Recognized As Safe (GRAS), including, for example, spores' preparations intended to be used as probiotic in several food types (Konuray & Erginkaya, 2018b; Lee et al., 2009). The use of *Bacillus* species as probiotics and the respective results have been described as strain specific (Lee et al., 2019). Despite being a less studied species as probiotic, previous studies have reported their effect in reducing total cholesterol, LDL‐c, and total/HDL‐c or LDL‐c/HDL‐c ratios (Angelino et al., 2019; Mazruei Arani et al., 2019; Mohan et al., 1990). Other trials demonstrated that a synbiotic composed by *Lactobacillus sporogenes* (currently designated by *Bacillus coagulans*) and inulin decreased TG and VLDL‐c, with no effect on total cholesterol, LDL‐c, and HDL‐c levels, in pregnant women (Taghizadeh et al., 2014) but with an increase in HDL‐c in diabetic patients (Shakeri et al., 2014). Our results are in line with some of these previous reports as we could observe that the synbiotic bread used in our intervention significantly decreased total cholesterol compared to control. A similar synbiotic (*L. sporogenes* and inulin) was used in a trial with diabetic patients and, after 6 weeks, no significant changes were observable in most lipid parameters, with only a slight change in TG and HDL‐c levels (Asemi et al., 2014).

Inulin is a prebiotic that was included in the synbiotic bread used in this trial. It is a soluble fiber present in several plants being usually included in regular diets. Several physiological roles have been associated with inulin including, for example, the reduction of glycemia and plasma blood lipids, and the promotion of minerals' and vitamins' absorption (Wan et al., 2020). Moreover, it has already been demonstrated that inulin is fermented by intestinal bacteria with the production of butyrate and other SCFA (Rossi et al., 2005; Wan et al., 2020). These SCFA have been shown to have several important metabolic roles (Wan et al., 2020) and, in particular, butyrate has been associated, in an animal model, to significant changes in liver TG, VLDL‐c, and bile acids' metabolism (Trautwein et al., 1998).

Inulin has been used in trials that aimed to evaluate its effect, ingested alone or in combination with different probiotics, showing positive health effects. For example, it has been reported that inulin may contribute to increase the relative abundance of *Bifidobacterium*, a beneficial component of gut microbiome (Reimer et al., 2020), and that, in combination with *L. acidophilus*, *Lactobacillus casei*, and *Bifidobacterium bifidum*, may decrease TC, TG, and LDL‐c, after 8 weeks, in overweight and obese subjects (Hadi et al., 2019). These previous results support our findings in this trial as “synbiotic bread,” containing inulin, induced positive changes in TC, TG, and Apo A1. The observed changes may be due to both *B. coagulans* and inulin, as already observed in other studies, and so an additional group selected to ingest bread supplemented only with inulin could have been interesting to evaluate and to elucidate the possible effect merely due to inulin. In fact, recent studies carried out with both human subjects and with mice models have reported the beneficial effects of inulin. For example, Li et al. (2021) have reported that inulin induced a significant reduction of total cholesterol (TC), triglycerides (TG), and LDL‐c in diabetic patients (Li et al., 2021). Additionally, it was observed that the effect of inulin on the hepatic fatty acid profile, gut microbiome composition, hepatic enzyme expression and activity, and pro‐inflammatory markers was more pronounced in mice fed with high‐fat diets than in those fed with normal or low‐fat diets (Albouery et al., 2021; Bao et al., 2020). A reduction in triglycerides (TG) and hepatic steatosis was also observed in mice fed with Western style diet supplemented with inulin (Beisner et al., 2021). Despite the recent observation of these positive effects of inulin, the mechanisms responsible for them are still unclear, although it is proposed that the modulation of the gut microbiome composition may play a key role in the process.

The addition of lactic acid to bread has been shown to reduce gastric emptying and to reinforce the interactions between starch and gluten (protein) making more difficult the enzymatic starch digestion (Fardet et al., 2007; Stamataki et al., 2017). Our results show that the ingestion of the bread containing only lactic acid (corresponding to group II) induced a more significant decrease in Apo A1 level compared to group III (synbiotic bread), which may suggest that the concentration of lactic acid may be higher when it is added to bread dough compared to what may be produced by the lactic acid‐producing probiotic used. This may have resulted in a larger amount of resistant starch, available to be fermented by the intestinal gut or in a decrease in pH which may affect bacterial metabolism. Additionally, we observed that the combination of the synbiotic supplement with lactic acid (group IV) provoked a more pronounced decrease in the concentration of Apo A1 and a significant decrease of total cholesterol, compared to control bread, ingested by group I (Table 3). These results suggest that the amount of lactic acid should be carefully chosen to avoid this effect on lowering Apo A1, an apoprotein associated with HDL and beneficial effects regarding cardiovascular diseases. The determination of LDL/HDL ratio could add more information on the effect of the tested types of bread on lipid profiles of the participants in this trial.

As far as we know, this is the first trial testing the effect of a synbiotic bread (*B. coagulans* and inulin), with or without the addition of lactic acid, on plasma lipid profile and apoprotein levels of T2DM patients. A similar trial with T2DM patients was carried out by Shakeri et al. (Shakeri et al., 2014) also using synbiotic bread (*L. sporogenes* [current *B. coagulans*] and inulin) during 8 weeks, but with the incorporation of beta‐glucan or lactic acid. Our trial presents some limitations that should be considered in future studies. Although we could observe a positive effect on total cholesterol, this was a relatively short trial (8 weeks). A recent systematic review has shown that the effect of synbiotics on lipid profiles is most significant in studies longer than 8 weeks (Hadi et al., 2020). In terms of the effect on gut microbiome, it would have been interesting to analyze the feces of the participants, in the beginning and the end of the intervention, to determine the main bacteria species and the amount of short‐chain fatty acids (SCFA), as it has been known that synbiotic have notable effects on these parameters that, in turn, are important to the control of plasma lipid levels. Additionally, an evaluation of the baking process in terms of its effect on the *B. coagulans* activity and on the rate of starch bioavailability, considering the addition of beta‐glucan, inulin, and/or lactic acid to the bread dough, could give a better understanding about the synbiotic mode of action. Moreover, we could not evaluate daily or weekly changes of the variables due to limited evaluation tools.

Nevertheless, this trial also had several strengths. Using bread as a vehicle to synbiotic supplements is a good choice considering that it is a type of food that is present in most diets, and which is consumed by most people. Choosing *Bacillus coagulans* as a probiotic to include in bread has the advantage of using a bacteria species that tolerates high temperature which is adequate to the baking process. It also tolerates acidic pH, making it suitable to use in the presence of lactic acid.

In addition, the incorporation of beta‐glucan, a well‐studied prebiotic with several beneficial health effects in all types of bread, including the control, was an additional advantage that allowed the evaluation of the effects of lactic acid and/or the synbiotic bread in comparison with a control that possesses cholesterol‐lowering ability and potentiates the production of SCFA (Chen & Raymond, 2008; Velikonja et al., 2019).

In conclusion, this trial showed that the synbiotic bread (with or without lactic acid) promotes a decrease in total cholesterol and Apo A1 in diabetic patients when consumed daily for 8 weeks. Additional studies are needed to overcome the identified limitations of this and other similar trials to better understand the beneficial effects of synbiotic supplements and their respective mechanisms of action.

## ACKNOWLEDGMENT

None.

## CONFLICT OF INTEREST

The authors have no conflicts of interest to declare.

## Data Availability

Not applicable.
